# Dr. S. H. Lynde

**Published:** 1904-11

**Authors:** 


Dr. S. H. Lynde, of Buffalo, a graduate of the University of Buffalo, 1889, died at his home, October 25, 1904, aged 35 years.

